# The Rise and Need for Mobile Apps for Maternal and Child Health Care in China: Survey Based on App Markets

**DOI:** 10.2196/mhealth.9302

**Published:** 2018-06-08

**Authors:** Puhong Zhang, Le Dong, Huan Chen, Yanling Chai, Jianbo Liu

**Affiliations:** ^1^ Department of Women and Child Health The George Institute for Global Health at Peking University Health Science Center Beijing China; ^2^ Department of Epidemiology and Statistics School of Public Health Hebei Medical University Shijiazhuang China; ^3^ Department of Health Education School of Public Health Peking University Health Science Center Beijing China

**Keywords:** mHealth, health services, maternal-child, mobile apps, market research

## Abstract

**Background:**

Mobile health services are thriving in the field of maternal and child health in China due to expansions in the field of electronic health and the introduction of the two-child policy. There are numerous maternal and child health apps in computer stores, but the exact number of apps, number of downloads, and features of these apps is not known.

**Objective:**

This study aimed to explore the use of maternal and child health apps in Android and iOS app stores and to describe the key functional features of the most popular apps, with the purpose of providing insight into further research and development of maternal and child health mobile health products.

**Methods:**

The researchers conducted a search in the 3 most popular Android app stores (Tencent MyApp, Baidu Mobile Assistant, and 360 Mobile Assistant) and the iTunes App Store in China. All apps regarding family planning (contraception and preparing for pregnancy), pregnancy and perinatal care, neonatal care and health, and development for children under 6 years were included in the initial analysis. Maternal and child health mobile apps with predominant features of product marketing, children’s songs, animation, or games were excluded from the study. The 50 most frequently used apps in each of the Android stores as well as the iTunes store (a total of 78 deduplicated apps) were selected and downloaded for an in-depth analysis.

**Results:**

A total of 5276 Android apps and 877 iOS apps developed for maternal and child health care were identified. Of the 78 most frequently used apps, 43 (55%) apps focused on one stage of MCH care, mainly targeting child care (25 apps) and before pregnancy care (11 apps), whereas 35 (45%) of the apps covered 2 or more stages, most of which (32 apps) included both pregnancy and child care services. The app features that were commonly adopted by the popular apps were health education, communication, health status self-monitoring, a diary, reminders, and counseling. Within the app feature of “health status self-monitoring,” the researchers found 47 specific tools supporting activities such as pregnancy preparation, fetal heart monitoring, blood glucose and blood pressure monitoring, and doctor visits. A few apps were equipped with external devices (n=3) or sensors. No app with intelligent decision-support features to support disease management for conditions such as gestational diabetes and pregnancy-induced hypertension was found. A small number of apps (n=5) had a Web connection with hospital information systems to support appointment making, payments, hospital service guidance, or checking of laboratory results.

**Conclusions:**

There are thousands of maternal and child health apps in the Chinese market. Child care, pregnancy, and before pregnancy were the mostly covered maternal and child health stages, in that order. Various app features and tools were adopted by maternal and child health apps, but the use of internal or external sensors, intelligent decision support, and tethering with existing hospital information systems was rare and these features need more research and development.

## Introduction

In the 21st century, mobile health (mHealth), defined as the use of mobile phones and other wireless technology in health care, is a burgeoning field within public health [1]. On July 1 2015, the State Council of the people’s republic of China issued “the Guiding Opinions of the State Council on Actively Promoting the Internet + Action,” outlining the idea of promoting a new model of Web-based health care [2], with mHealth playing an increasing role in the delivery of health care. Improving the health and well-being of women and children has remained a common goal throughout the world [3-6].

With the rapid development of mHealth, thousands of maternal and child health (MCH) apps have appeared in China in Android app markets and Apple’s app market. The popularity of mobile internet use (through devices such as mobile phones) and the universal two-child policy [7] will most likely result in an expansion of the current app market on MCH care. The market research agency “iResearch” reported that the use of Chinese maternal and child mobile apps (excluding mobile business apps) increased at a rate of 40% and 60% in 2015 and 2016, respectively [8]. However, almost no research seems to focus on the development and functional features of the Chinese mHealth products on MCH care. Hence, we conducted a study intending to find the most frequently used apps on MCH care in Android and iOS app stores, describe the utility and features, and to provide recommendations for app developers and researchers.

## Methods

### Selection of App Markets

According to “The most popular apps for Androids” released by the *Internet Weekly* in 2016, *Tencent MyApp*, *Baidu Mobile Assistant,* and *360 Mobile Assistant* were the top 3 Android app markets in China [9]. Hence, we selected the 3 mainstream Chinese Android app markets and iOS application market iTunes App store to retrieve MCH-related apps.

### Selection of Maternal and Child Health Apps

We selected the apps developed for family planning (contraception and preparation for pregnancy), pregnancy and perinatal care, neonatal care and health, and development of children under 6 years. We did a preliminary search in the 4 app markets using the following keywords in the Chinese language: *pregnant, pregnancy, postpartum, child care, maternity, maternal and child, child, infant,* and *mother*. On the basis of the preliminary search results, a few other phrases frequently used in MCH app descriptions were added to the keyword searches, which were *menstruation, women, pregnancy stage, baby, fashion mother, fetus, mother and child, children’s songs,* and *early education.*

For each of the 4 app markets, the official website or App store was visited, and we searched for the MCH apps using the identified keywords. For each search, we logged in as a guest so that the search results could not be tailored to an existing account [10]. The app name and the number of downloads (Android apps) or the number of reviews (iOS apps) for all apps generated from a search of each keyword in each app market were recorded. Two blinded investigators screened the name and the description of the searched apps. The apps that were not relevant to MCH care and product marketing apps were excluded. App deduplication was conducted for Android and iOS apps separately. The number of downloads for the same Android app in different markets were summed to obtain the total downloads for the app. The search of the app stores was conducted between February 15 2017 and to March 1 2017.

### Selection of Frequently Used Apps for In-Depth Analysis

All found apps were ordered by number of downloads (Android) or reviews (iOS). The top 50 apps from Android and iOS markets respectively were deemed as the most frequently used apps and were selected for in-depth analysis in this study. A large number of apps were found which focused on early child education by predominantly using children’s songs, animation, and intelligence games. These apps had limited features or research significance, but the rankings were comparatively high. For our research purposes, we excluded these apps when identifying the top 50 apps in both markets.

For the apps that employed both Android and iOS systems, only the iOS system was used for in-depth analysis, as small differences could be identified between the different versions. Finally, the lists of the top 50 searches for Android and iOS were integrated into a combined list, which contained 78 unique apps.

Additionally, apps developed by, or mainly used by MCH institutions, were ordered by number of downloads (Android) and reviews (iOS), and the 10 most frequently used “top 10 institution apps” were selected for further analysis.

### In-Depth Analysis of Maternal and Child Health Apps

The 78 frequently used apps and the top 10 institution apps were all downloaded and installed on an iPhone 6s (iOS 10.3.1) or a Huawei MATE8 (NXT-AL10 Android 6.0) for in-depth analysis. For each app, 2 independent investigators registered and logged in to check all app modules and information. For the app or interventions that could only be activated at specific situations or stages, we activated them by simulating the required situations such as setting the expected date of confinement or fetal birth, registering multiple accounts, and adjusting the system time of the mobile phone.

To conduct the in-depth analysis, a semistructured database to collect and record the app characteristics was used. This database included the app’s name, the number of downloads or reviews, MCH stages, mHealth app features, specific interventions or services, and the mobile phone functions. Specifically, the MCH stages were classified as before pregnancy, pregnancy, birth, postpartum (mother), and child care (covering postnatal newborn, infancy, and childhood). The mHealth app features we intend to analyze comprise of 3 key components, namely (1) a list of 12 common app features mainly targeting health care providers, (2) a list of 5 app features serving client users, and (3) a list of 4 app features observed in the identified apps during the in-depth analysis. The first list of app features was based on a 12-category framework evaluating health systems performance for mHealth innovations in MCH field [11]. They are client education and behavior change communication (subdivided into health education and counseling in this study), sensors and point-of-care diagnostics, registries and vital events tracking, data collection and reporting, electronic health records, electronic decision support, provider-to-provider communication, provider work planning and scheduling, provider training and education, human resource management, supply chain management, and financial transaction and incentive [11]. The second list of app features came from a study which added amendments to the 12-category framework and included health status self-monitoring (expanded from the name of physical or bio data monitoring to cover social and psychological status evaluation, could be facilitated by specific sensors or standardized assessment scales), reminders, appointment making, client-to-client communication, and laboratory result checks [12]. The third list of app features, which were observed during the in-depth analysis, included client diary, shopping, games, and hospital service promotion (including introduction of hospitals, departments, doctors, and hospital events as well as hospital navigation or intelligent guidance).

## Results

### Characteristics of Identified Apps

**Figure 1:**
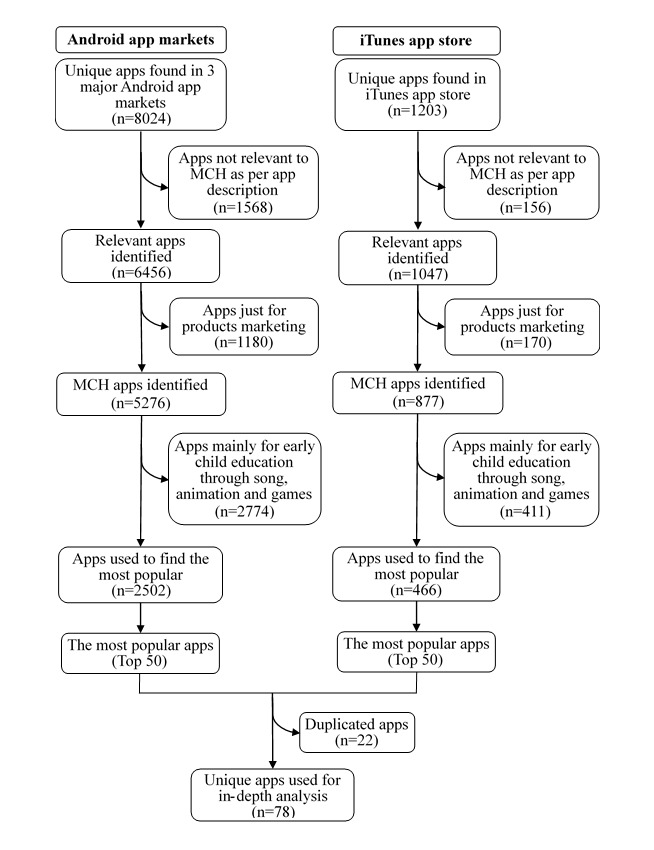
Flowchart of app selection for maternal and child health. MCH: maternal and child health.

A total of 5276 Android system MCH apps and 877 iOS system MCH apps were identified. The flowchart of MCH app selection is shown in Figure 1. Table 1 shows the distribution of the identified MCH apps with different numbers of downloads or reviews.

After excluding apps whose predominant feature was early child education through songs, animation, and games (n=2774), the top 50 apps from Android and iOS app markets separately were selected, and 78 deduplicated apps were regarded as the most frequently used ones and downloaded for analysis.

**Figure 2:**
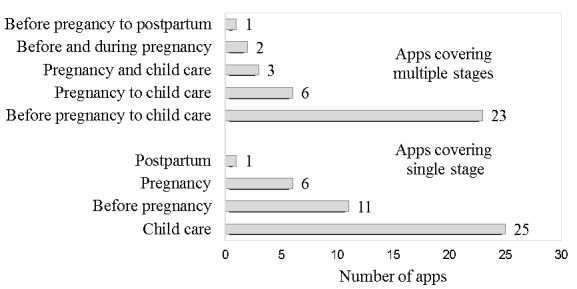
Stages of maternal and child health care covered by the 78 most popular apps.

All of the 78 frequently used apps used in this study were developed by private companies and were available for free download and use. Each app had over 2,570,000 downloads or more than 1235 reviews. For *Meet You*, an MCH app developed by Xiamen Mei Pomelo Information Technology Co, Ltd, the cumulative number of downloads exceeded 100 million since it was put into use in 2013. As shown in Figure 2, more than half of the frequently used apps (43/78, 55%) focused on only 1 stage, and nearly half (35/78, 45%) covered 2 or more stages of MCH care. For the apps that had covered only 1 stage, child care was most targeted, followed by before pregnancy (family planning); for those targeting 2 or more stages, the entire range of MCH care (ie, from family planning to child care), or nearly the entire range (ie, from pregnancy to child care), were covered.

### Features of the Frequently Used Apps

**Figure 3:**
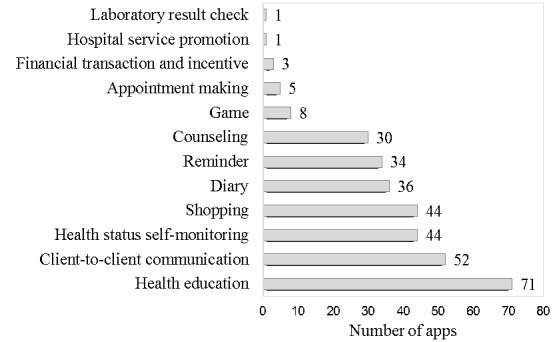
App features adopted in the 78 most popular apps on maternal and child health care.

The app features adopted by the 78 frequently used apps are illustrated in Figure 3. Health education (71/78, 91%), client-to-client communication (52/78, 67%), health status self-monitoring (44/78, 56%), shopping (44/78, 56%), diary (36/78, 46%), reminders (34/78, 44%), and counseling (30/78, 39%) were among the most commonly developed app features. A handful of apps (n=5) had links to existing hospital information systems to support appointment making, payment, hospital service promotion, and to check laboratory results.

**Figure 4:**
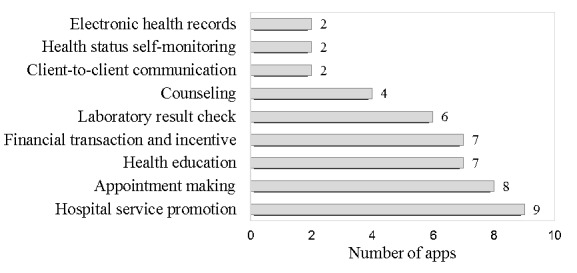
App features covered in apps developed by maternal and child health institutions.

The top 10 MCH institute apps developed for maternal and child health organizations were also screened for and analyzed. They had, at most, 18,000 downloads and no more than 39 reviews. The main app features were hospital service promotion, appointment making, health education, financial transaction and incentive (only payment for service), checking laboratory results, and counseling. Figure 4 shows the detailed features of these apps.

### Specific Tools Supporting Health Status Self-Monitoring

Within the “health status self-monitoring” app feature, 47 specific tools were found to provide users with individualized feedback as a response to regular or occasional health data collection (Table 2). The tools were designed to provide certain support on pregnancy preparation, fetal heart monitoring, blood glucose and blood pressure monitoring, and some action reminders. Three apps were equipped with external sensors or devices to monitor basal body temperature, body weight, physical exercise, fetal movement, and contractions. One app adopted intelligent technology to monitor heart rate through “fingertip scanning” using a phone camera. No intelligence support tools or app features in the studied apps to support electronic and smart management of diseases such as gestational diabetes and pregnancy-induced hypertension were found.

**Table 1:** Distribution of maternal and child health care apps with different number of downloads or reviews.

Frequently used apps		n (%)
**Android apps, number of downloads**		**5276 (100)**
	≥50,000,000	8 (0.15)
	≥10,000,000	40 (0.76)
	≥5,000,000	40 (0.76)
	≥1,000,000	219 (4.15)
	≥500,000	148 (2.81)
	≥100,000	488 (9.25)
	≥50,000	303 (5.74)
	≥10,000	781 (14.80)
	≥5000	411 (7.79)
	≥1000	1074 (20.36)
	<1000	1764 (33.43)
**iOS apps, number of reviews**		**877 (100)**
	≥100,000	3 (0.3)
	≥50,000	3 (0.3)
	≥10,000	24 (2.7)
	≥5000	25 (2.9)
	≥1000	96 (10.9)
	≥500	64 (7.3)
	≥100	197 (22.5)
	≥100	465 (53.0)

**Table 2:** Specific tools within the “health status self-monitoring” feature found in the 78 popular apps on maternal and child health.

Tools		Apps, n (%)
**Planning for pregnancy**		
	Menstruation	21 (27)
	Sex life	16 (21)
	Ovulation	13 (17)
	Body symptoms	13 (17)
	Basic body temperature	13 (17)
	Leucorrhea	7 (9)
	Defecation	4 (5)
	Folic acid	3 (4)
	Type-B ultrasonic to test ovulation	2 (3)
	Sleep	2 (3)
	Medication	2 (3)
**Pregnancy stage**		
	Fetal movement	13 (17)
	Antenatal examination	10 (13)
	Expected date of confinement	10 (13)
	Abdominal girth perimeter	10 (13)
	Biparietal diameter	9 (12)
	Femur length	9 (12)
	Uterine contraction	8 (10)
	Antenatal examination report	2 (3)
	Blood pressure	2 (3)
	Fetal heart	2 (3)
	Parents blood type	2 (3)
	Progesterone value	1 (1)
	Human chorionic gonadotropin value	1 (1)
	Blood glucose	1 (1)
	Fundal height	1 (1)
**Postpartum stage**		
	Postpartum depression	1 (1)
**Parenting stage**		
	Children’s height	18 (23)
	Children’s weight	18 (23)
	Vaccination	11 (14)
	Children’s head circumference	9 (12)
	Nurse	5 (6)
	Children’s sleep	5 (6)
	Children’s defecation	4 (5)
	Supplementary food	3 (4)
	Children taking drugs	2 (3)
	Children’s body temperature	1 (1)
**Before and during pregnancy**		
	Exercise	9 (12)
	Diet	7 (9)
**All stages**		
	Body weight	23 (29)
	Body height	11 (14)
	Body mass index	3 (4)
	Medical advice	2 (3)
	Medical records	2 (3)
	Heart rate	1 (1)
	Users’ blood type	1 (1)
	Laboratory test report result	1 (1)

## Discussion

### Principal Findings

This study revealed that there is a large demand for MCH mobile apps in the Chinese market. The cumulative downloads for all MCH apps amounted to hundreds of millions. When considering the stage of MCH which the developed apps targeted, child care was the most covered stage if the app was developed to target only 1 stage or the whole stage from (before) pregnancy to child care were the most covered stages when the apps were developed. Health education, communication, health status self-monitoring, shopping, diary, reminders, and counseling were the most developed app features. With respect to the specific app feature, “health status self-monitoring,” there were 47 tools helping women through family planning to child care. However, very few apps had effective communication between market MCH apps and existing hospital information systems, and very few apps were equipped with external or internal sensors or devices to support prompt data collection and point-of-care diagnostics.

### Applications Adopted

Given that the target population of the most frequently used apps was the general public , the application features facilitating professional staff providing health service management and reporting were rarely adopted. These features included registries and vital events tracking, data collection and reporting, electronic health records, electronic decision support, provider-to-provider communication, provider work planning and scheduling, provider training and education, human resource management, supply chain management, and financial transaction and incentive. Instead, health education, personalized reminders, health status self-monitoring, counseling and client-to-client communication were the most adopted app features.

mHealth has large potential in health education activities due to its effectiveness in delivering verbal and visual messages [13]. More than 90% (71/78) of the frequently used apps had adopted health education app features. According to social cognitive theory, individuals would gain better understanding and learn quickly from observing and are likely to remember and repeat the behaviors provided by a model [14,15]. App developers could strengthen health education activities by improving user interface design to include more pictures, scene animation, and video information which is more easily understood by a wider range of people.

Personalized reminders can reinforce behavioral changes in app users [16,17]. In the studied apps, the reminders were customized to the specific health status of users and included the events such as antenatal examinations, vaccinations, drinking water, taking drugs, etc. However, the reminders were of varying quality; in many cases, further improvement and standardization are needed.

Health status self-monitoring tools are often a means to track changes in physical, biological, social, and psychological indicators, which in turn can guide corresponding behavior change [18]. In this study, dozens of tools were found (Table 2). Some tools were used quite frequently in certain MCH stages; however, further studies are needed to explore the validity and effectiveness of these tools.

Counseling is also a widely adopted app feature among the studied MCH apps. It can be a convenient way to connect users and experts, including either real-time consultations or nonreal-time queries through text, voice, and picture messages. This feature could facilitate, if adopted properly, education, medication instruction, and appointment scheduling. However, it must be noted that all the counseling behaviors should comply with the local regulations and laws.

Client-to-client communication could meet the desire of users to seek peer support by communicating with people who had similar health issues. Many apps would assign some pregnant women who are experienced in receiving health service with the support of app to answer questions in order to enhance the interaction. In fact, communication has been determined as an important measure to ensure the success of an app [19].

Other supportive features such as diaries, shopping, and games, which were not key components of MCH care but were highly welcomed by users, had a large impact on users’ adherence to apps. A diary can help people to record their mood and psychological status and experience being a new mother; shopping features can help people finding food, clothes, and other daily necessities good for health; and games might attract continuing use of an app. More and more apps now have features that promote what to eat and where to shop; however, this would have a negative impact if the app gives improper advice regarding food, nutritional products, and other daily necessities. The shopping app feature is the main profit model of current MCH apps in the Chinese app markets and needs further improvement.

Some apps were trying to connect with the MCH units in hospitals through features on clinical support services, namely appointments, payments, test results, etc; however, the relative downloads of apps with these features were much lower. Currently, most hospital information systems are closed networks. It is challenging to achieve free data transmission between apps and existing hospital electronic systems, given the concern over safety issues. Another attempt to connect with medical system was to develop MCH hospitals’ own apps. Such apps focused on hospital service promotion, appointment making, and checking of laboratory results. Due to the lack of commercial running, most of the apps were not well developed or maintained.

### Limitations

The study retrieved 5276 and 877 MCH apps, respectively, in the Android market and iTunes App store by using the most comprehensive keywords. However, not all apps were recruited, given that an increasing number of apps were developed on WeChat (a communication app of Tencent), and we did not include them in the study due to its unique interface and app structure. In addition, we are not able to backtrack the dates of release for all the studied apps. The difference on survival time will have an influence on the number of downloads and reviews. As a result, this may cause bias when identifying the most frequently used apps based on the number of downloads or reviews of the apps.

**Conclusions**

MCH apps have been rising in China’s market. Most of the apps were equipped with various features and tools. This study may provide an insight on the selection of appropriate features, functions, and tools and may facilitate a better understanding for mobile app developers of the gaps existing in mHealth products.

## Abbreviations

MCH: maternal and child health
mHealth: mobile health
